# Variations in the identity and complexity of endosymbiont combinations in whitefly hosts

**DOI:** 10.3389/fmicb.2014.00310

**Published:** 2014-07-04

**Authors:** Einat Zchori-Fein, Tamar Lahav, Shiri Freilich

**Affiliations:** ^1^The Agricultural Research Organization, Newe Ya'ar Research Center, Institute of Plant ProtectionRamat Yishay, Israel; ^2^The Agricultural Research Organization, Newe Ya'ar Research Center, Institute of Plant SciencesRamat Yishay, Israel

**Keywords:** bacteriome, *Bemisia tabaci*, facultative endosymbionts, holobiont

## Abstract

The target of natural selection is suggested to be the holobiont - the organism together with its associated symbiotic microorganisms. The well-defined endosymbiotic communities of insects make them a useful model for exploring the role of symbiotic interactions in shaping the functional repertoire of plants and animals. Here, we studied the variations in the symbiotic communities of the sweet potato whitefly *Bemisia tabaci* (Hemiptera: Aleyrodidae) by compiling a dataset of over 2000 individuals derived from several independent screenings. The secondary endosymbionts harbored by each individual were clustered into entities termed Facultative Endosymbiont Combinations (FECs), each representing a natural assemblage of co-occurring bacterial genera. The association of FECs with whitefly individuals stratified the otherwise homogeneous population into holobiont units. We both identified bacterial assemblages that are specific to whitefly groups sharing unique genetic backgrounds, and characterized the FEC variations within these groups. The analysis revealed that FEC complexity is positively correlated with both distance from the equator and specificity of the genetic clade of the host insect. These findings highlight the importance of symbiotic combinations in shaping the distribution patterns of *B. tabaci* and possibly other insect species.

## Introduction

The term “holobiont” was coined to describe a central, multi-cellular organism and all of its associated symbiotic microbes, including parasites, mutualists, synergists, and amensalists (Rosenberg and Zilber-Rosenberg, 2011). Variations in the structure and composition of holobiont communities have been shown to affect the fitness of the host, suggesting that the target of natural selection is not only the organism itself but also its associated community of microorganisms (Rosenberg et al., 2007). Simple symbiotic systems such as those formed between many arthropods and a limited-size community of bacterial endosymbionts (i.e., bacteria that reside within the cells of their hosts, as opposed to gut bacteria for example) can provide useful models for exploring the functional variations among holobiont units (Ferrari and Vavre, 2011).

Many insects, mainly those feeding on a nutrient-imbalanced diet (e.g., plant sap, wood, or vertebrate blood), possess a specialized organ termed bacteriome that hosts endosymbiotic bacteria (Buchner, 1965; Baumann, 2005). Within the bacteriome, insect cells provide nutrients and shelter for their tenants; in exchange, bacteria, that are classified as “primary” or “obligated” endosymbionts, complement the insect's diet by providing essential and otherwise missing nutrients such as vitamins, amino acids and carotenoids (McCutcheon and Moran, 2010; Sloan and Moran, 2012; Russell et al., 2013). Such obligatory endosymbionts are strictly maternally inherited, their phylogeny is congruent with that of their host and they tend to be fixed in insect species, genera and families (Zchori-Fein and Bourtzis, 2011). Alongside the primary endosymbionts, insects often carry non-essential bacterial associates termed “facultative” or “secondary” endosymbionts that can also be housed within the bacteriome, as well as in various other tissues (Zchori-Fein and Bourtzis, 2011). Unlike the primary endosymbionts, facultative associates are transmitted not only vertically, through maternal inheritance, but also through occasional events of horizontal transmission. Such transfer, spreading and loss events lead to dynamic variations in bacterial communities within insect populations. These variations are suggested to form a “horizontal gene pool” which may provide fitness benefits to the insect host (Moran, 2007; Gueguen et al., 2010; Jaenike, 2012; Henry et al., 2013). Studying the ecological factors that are associated with such variations can point at the functional significance and the selective pressures that promote the sustainability of different holobiont units assembled around a single central species.

In the past decade, the emergence of new genomic sequencing techniques has allowed the characterization of specific microbial communities across significant number of insect individuals. Two recent surveys of symbionts associated with aphids (Henry et al., 2013) and whiteflies (Gueguen et al., 2010) individuals have pointed at a dynamic process of community assemblage resulting from horizontal transfers and reflecting ecological similarities (e.g., host plant) among individuals carrying specific symbionts. The symbiotic communities of the phloem-sap feeding sweetpotato whitefly *Bemisia tabaci* (Hemiptera: Aleyrodidae)—a major pest of several key crops worldwide (Stansly and Steven, 2010)—have been extensively documented. *B. tabaci* is a species complex consisting of as many as 34 genetically distinct but morphologically indistinguishable, delimited genetic groups (De Barro et al., 2011; Liu et al., 2012; Tay et al., 2012). Most of these genetic groups are equivalent to the “biotypes” of earlier works, and for the sake of simplicity, we will use this definition hereafter. Like the entire Aleyrodidae family, all *B. tabaci* individuals carry the primary endosymbiotic bacterium *Portiera aleyrodidarum* (Thao and Baumann, 2004). Frequently, individuals also carry varying combinations of one to four facultative endosymboints out of seven bacterial genera (*Wolbachia, Cardinium, Rickettsia, Arsenophonus, Hamiltonella, Fritschea*, and *Hemipteriphilus*). Occurrence patterns of facultative endosysmbionts have been attributed to several aspects of the insect's biology, including host reproduction (Zchori-Fein et al., 2001; Hunter et al., 2003; Zchori-Fein and Perlman, 2004; Himler et al., 2011), survival and fecundity (Liu et al., 2007; Kontsedalov et al., 2008; Gottlieb et al., 2010; Thierry et al., 2011), resistance to insecticides (Kontsedalov et al., 2008) and capacity to transmit diseases to the host plant (Gottlieb et al., 2010). Variations *among* biotypes in their association patterns with facultative endosymbionts are repeatedly reported (e.g., Chiel et al., 2007; Gueguen et al., 2010). In previous screening efforts, the strong biotype-endosymbiont associations impede the characterization of additional, possibly more subtle determinants of facultative endosymbiont distribution, beyond biotype identity. In order to delineate possible environmental factors that are associated with community variations we compiled the results from several independent screens, reporting the distribution patterns of facultative endosymbionts across over 2000 *B. tabaci* individuals. Taking advantage of environmental diversity of the sampling sites, we used this collection for characterizing the diversity of bacterial assemblages *within* genetic groups.

## Materials and methods

### Database

**Figure 1:**
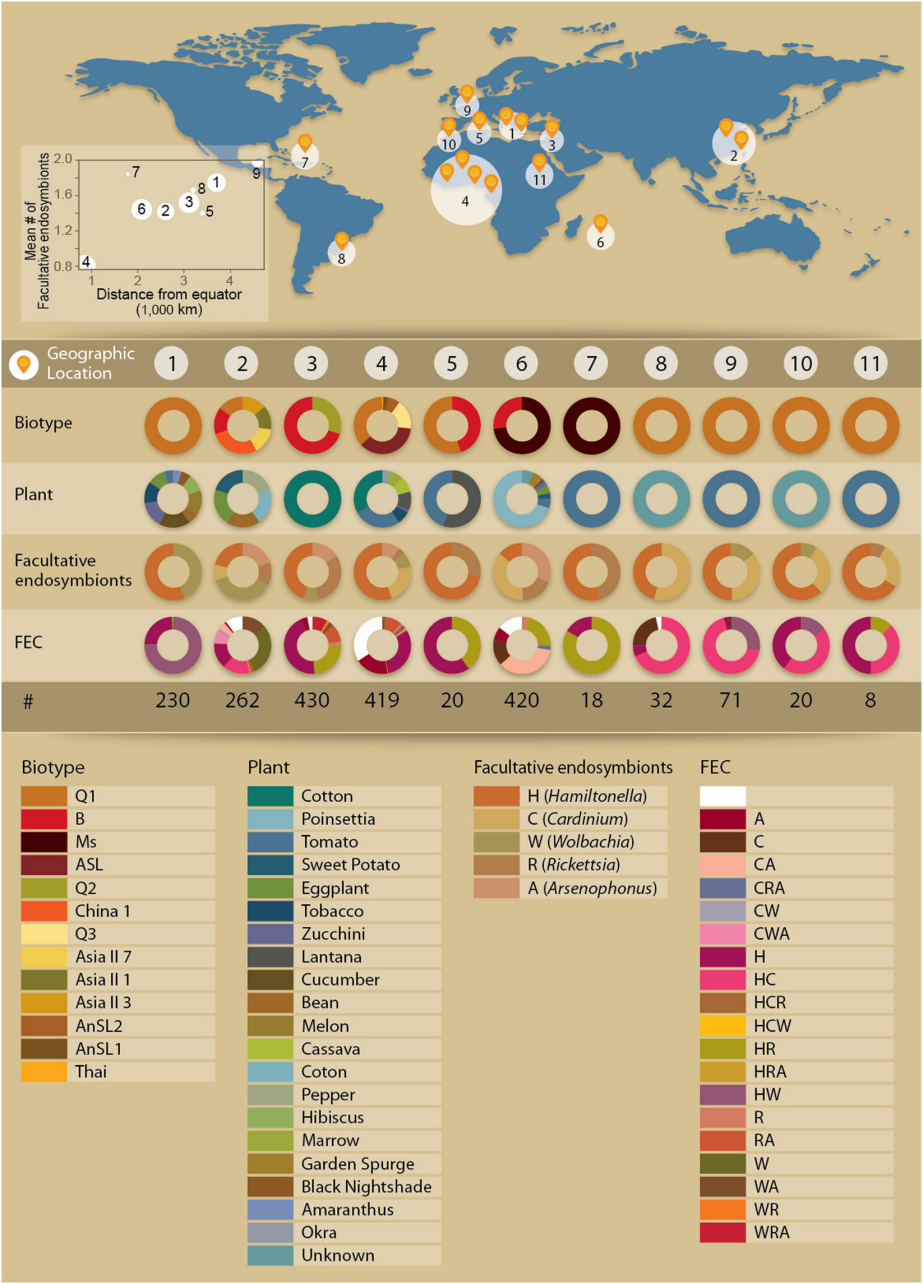
**Description of the dataset. Top**: Collection sites of *B. tabaci* included in the dataset. In some cases, small samples from proximal locations were grouped (e.g., location 4 in West Africa). The plot on the left shows the mean genera richness of facultative endosymbionts co-infecting whitefly individuals in each location (Pearson correlation: 0.65, *P*-value 0.03). Circle sizes indicate the number of sampled individuals. **Bottom**: Distribution of *B. tabaci* biotypes, plants, and facultative symbionts in each geographical location. FS, facultative symbiont; FEC, facultative symbiont combination; #, sample size. Scientific names of the host plants: cotton (*Gossypium* sp.), poinsettia (*Euphorbia pulcherrima*), tomato (*Solanum lycopersicum)*, sweet potato (*Ipomoea batatas*), eggplant (*Solanum melongena)*, tobacco (*Nicotiana tabacum*), zucchini (*Cucurbita pepo* var. *cylindrica*), lantana (*Lantana* sp.), cucumber (*Cucumis sativa*), bean (*Phaseolus vulgaris*), melon (*Cucumis melo*), cassava (*Manihot esculenta)*, pepper (*Capsicum annuum*), croton (*Codiaeum variegatum*), hibiscus (*Hibiscus mutabilis*), marrow (*Cucurbita pepo* var. *pepo*), garden spurge (*Euphorbia* sp.), black nightshade (*Solanum nigrum*), amaranthus (*Amaranthus retroflexus*), and okra (*Abelmoschus esculentus*).

Information from six worldwide screening projects of bacterial abundance within *B. tabaci* was combined to obtain the most comprehensive database to date in terms of the number of individuals sampled and the geographical range of their habitats. Overall, individuals were sampled from 20 host plants across 11 geographical locations that fall into 7 climatic zones and are classified into 13 distinct biotypes (Figure 1). The numbers of individuals sampled in the independent projects were: Project A: 330 (Tsagkarakou et al., 2012); Project B: 262 (Bing et al., 2013); Project C: 430 (Zchori-Fein's lab, unpublished); Project D: 237 (Gueguen et al., 2010); Project E: 393 (Thierry et al., 2011); and Project F: 378 (Gnankine et al., 2012). The biotype classifications of individuals from each project and the endosymbiont they harbor are provided at the Supplementary Material. All laboratories applied almost identical protocols for species' detection, detailed at the Supplementary Material. Briefly, biotype recognition generally relies on sequence-based phylogenetic analyses using numerous sequences of the mitochondrial gene cytochrome oxidase I (COI) (Boykin et al., 2007). The presence of *Portiera* was determined as an internal control for DNA quality. All individuals were screened for the presence of five facultative endosymbionts (*Arsenophonus, Cardinium, Hamiltonella, Rickettsia*, and *Wolbachia*) using PCR primers targeting the 16S rRNA gene for *Cardinium, Hamiltonella*, and *Rickettsia*, the 23S rRNA gene for *Arsenophonus* and the wsp gene or the 16S rRNA gene for *Wolbachia* (Supplementary Material). *Fritschea* and *Hemipteriphilus* were not screened by all groups and hence were not considered in the analysis. We verified that all screens allow the identification of each of the five endosymbionts by counting the number of individuals from a specific biotype carrying a specific endosymbiont in each project (Supplementary Materials). All five endosymbionts were identified in three of the projects (projects B, D, F); four out of five endosymbionts were identified in two of the projects (projects C, E); and in a single project, three out of five endosymbionts were identified (project A). Overall, out of 30 laboratory procedures of endosymbiont identification (5 endosymbionts X 6 projects), only four procedures did not result in the identification of an endosymbiont (Supplementary Material). Three out of these four procedures can be explained by the biotype identity of the individuals screened in the project: (1) *Cardinium* was not identified in project (C), but only individuals from B and Q2 were screened and *Cardinium* was not detected in these biotypes in any of the other projects. (2) *Wolbachia* was not identified in project (E), but only individuals from Ms and B were screened and *Wolbachia* was not detected in these biotypes in any of the other projects. (3) *Arsenophonus* was not identified in project (A), but only individuals from Q1 were screened and *Arsenophonus* was not detected in that biotype in of the other projects. Hence, though the screens were carried independently by different groups, the use of a common procedures and the consistency of the variations in the endosymbiont-biotype associations across projects (Supplementary Materials) support the integration of the data and its further use for delineating intra-biotype variations.

### Statistical analyses

The probability that the number of individuals classified to a given biotype and harboring a specific Facultative Endosymboint Combination (FEC) will be collected from a given host plant or geographical location was higher than chance, was determined by calculating a cumulative hypergeometric *P*-value. The corresponding size of the population was calculated as the sum of individuals collected for a given biotype; the number of items with the desired characteristic in the population, K, was the number of individuals in the population carrying a given FEC, and number of samples drawn, N, was the number of individuals in the population that were collected on a given host plant or in a given geographical region. Cumulative hypergeometric probability and partial correlations were calculated using Matlab.

## Results

### Characterization of infection richness of whitefly individuals with facultative endosymbionts

Information on the rate of occurrence of key facultative endosymbionts was collected by compiling data derived from independent projects. The analysis of the combined dataset reinforced past phylogenetic reports, derived from smaller-scale screens, which have delineated patterns of non-random occurrence of facultative endosymbionts among well-resolved *B. tabaci* genetic clades (Supplementary Materials). *Hamiltonella* is abundant in biotypes B and Q1 and *Arsenophonus* in other biotypes, mainly Ms and Q2; *Wolbachia* was not detected in individuals from biotype B, despite the large number of whiteflies included in the dataset. Most individuals were found to harbor at least one facultative endosymbiont (88%), with nearly half of the individuals surveyed (49%) harboring more than one, in agreement with previous observations (Gueguen et al., 2010). Analysis of the mean infection richness (number of genera) per individual across the different locations showed that community complexity increases with distance from the equator (Figure 1). We verified that this geographical gradient of infection richness does not reflect biases introduced by sensitivity differences between laboratory equipment and protocols by further grouping whitefly individuals not only according to their geographical locations but also according to their contributing groups. Reassuringly, high similarity in the mean infection richness among populations collected at a common geographic location and analyzed by different groups was observed (Supplementary Material). Similarly, we observe a richness gradient in *B. tabaci* populations collected at different locations and analyzed by a single group.

### Characterization of facultative endosymbiont combinations (FECs) and their distribution across biotypes

**Figure 2:**
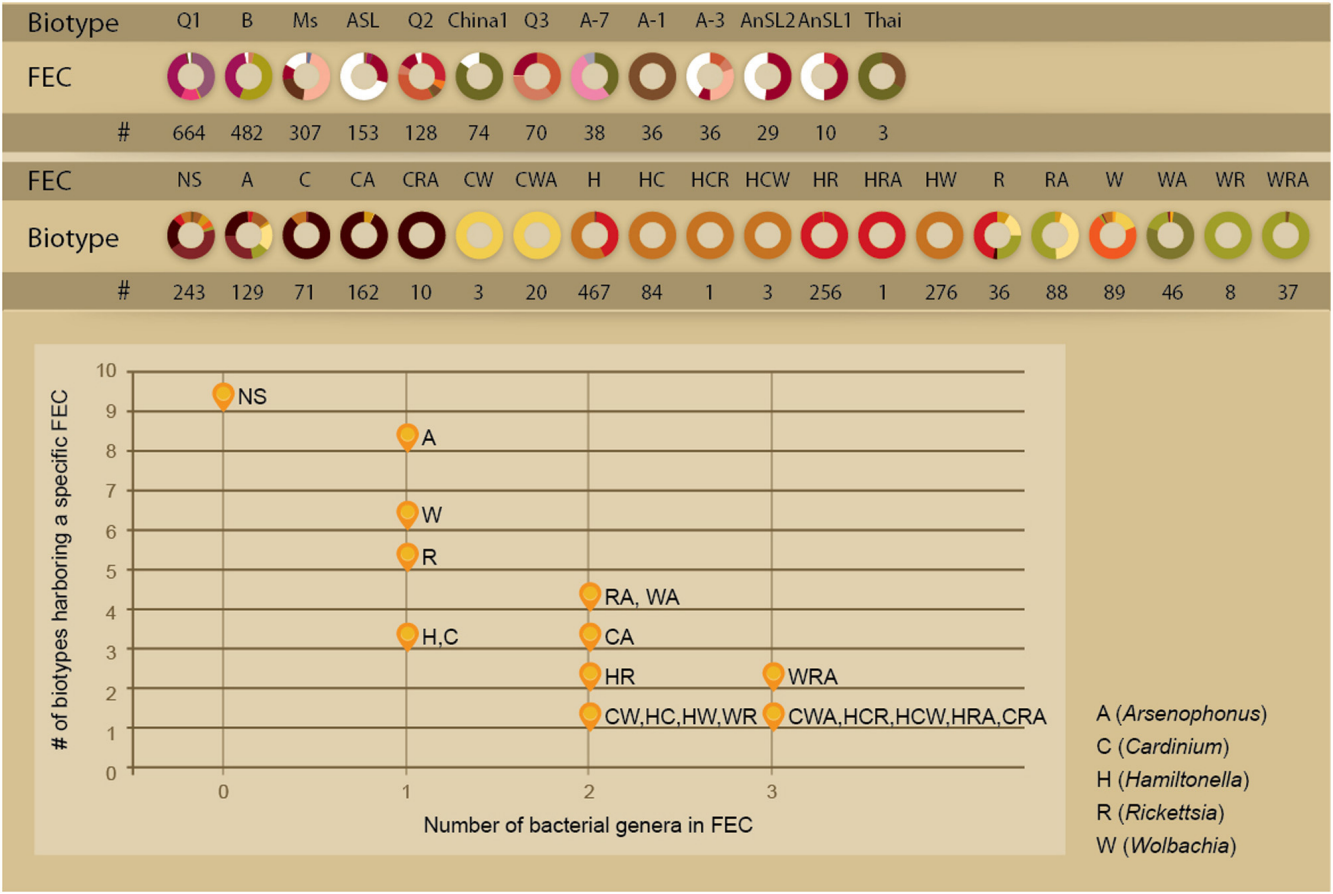
**Distribution of facultative endosymbiont combinations (FECs) in biotypes. Top:** Biotype distribution of FEC and the FEC association of biotypes. Colors are as in Figure 1. **Bottom**: Number of biotypes associated with a FEC entity as a function of its genera richness. The correlation remains significant after controlling for the number of whitefly individuals carrying a FEC entity. (Pearson: 0.7708, *P*-value 6e–5). A-7/1/3 biotypes stand for Asia II 7/1/3, respectively. NS, no symbionts; FEC, facultative endosymbiont combination; #, sample size.

The analysis of the factors associated with community variations is hampered by the complexity of the ecological system: the insects that host the endosymbionts are classified into several genetic clades and were collected from different host-plants at different locations (Figure 1). Individuals might harbor none, single or several facultative symbionts forming a range of possible combinations. To gain a community perspective and to simplify the association analyses by reducing the number of factors analyzed, we clustered all of the facultative symbionts harbored by an individual insect into entities termed Facultative Endosymbiont Combinations (FECs), each representing a natural assemblage of microbial genera that co-occur within distinct boundaries. Nineteen such FEC entities were detected across the database, including five, eight, and six combinations of single-, two- and three-genera combinations, respectively (Figure 2). FEC are termed according to the initials of their genera members (A, C, H, R, and W represent *Arsenophonus, Cardinium, Hamiltonella, Rickettsia*, and *Wolbachia*, respectively) where the number of letters represents the number of genera members. That is, FEC H represents a single member combination grouping together individuals for which *Hamiltonella* is the only facultative endosymbiont detected; FEC HW represents a two-members combination grouping together individuals for which *Hamiltonella* and *Wolbachia* are the only facultative endosymbionts detected. A strong negative correlation between FEC complexity (number of genera) and the number of biotypes harboring that specific FEC was observed (Spearman's Rho −0.83, *P*-value 5e–6, Figure 2). These results clearly show that the FEC–biotype association was stronger for multi-member combinations than for single-member ones, with most multi-member combinations being harbored exclusively by a single biotype. For example, as a single-genus combination, *Hamiltonella* (FEC H) was the only bacterium in the dataset that could be found in both Q1 and B biotypes. In contrast, the distribution ranges of H-containing multi-member communities were limited to either the Q1 biotype (combinations HC, HW, HCR, and HCW) or the B biotype (combinations HR and HRA) (Figure 2). Similarly, 62% of the FEC C (*Cardinium* only combination) were found in the Ms biotype. The association of C with this biotype was more pronounced when that bacterium was part of multi-member FECs; 90 and 83% of the CA combinations (CA and CRA, respectively) were harbored by the Ms biotype; CW combinations (CW, CWA) were limited to the Asia II 7 biotype, and HC combinations (HC, HCR) were limited to the Q1 biotype (Figure 2).

### Characterization of the intra-biotype diversity in FECs

Although previous screening studies have indicated that biotypes differ in their association patterns with facultative endosymbionts, the current analysis reports also an intra-biotype diversity of associated FECs. With the exception of Asia II 7, all biotypes harbor multiple FECs (up to 9, Figure 2). Overall, 58 unique biotype-FEC associations were identified in the dataset. The biotype-FEC entities stratify an otherwise homogeneous population (a biotype) into different holobiont units. To explore the functional significance of alternative combinations associated with a single biotype, we compared the plant and geographical preferences associated with the presence of a specific FEC. Focus was placed on B and Q1 insect individuals, as these were the two most extensively sampled biotypes across diverse locations (Supplementary Materials). Because the other key biotypes sampled (Ms, ASL, and Q2) were collected in a single geographical location with an almost exclusive host plant, the data did not allow carrying out a comparative analysis (Figure 2).

The distribution of a specific FEC-carrying individuals from biotypes Q1 and B across 9 and 5 geographical locations, and 15 and 6 host plants, respectively, is shown in Table 1. The distribution of Q1-associated FECs across host plants revealed that Q1_H_ (Q1-biotype individuals harboring *Hamiltonella* only—the FEC “H”) was sampled from cotton at a significantly higher frequency than excepted by chance, considering the frequency of H FEC in Q1 individuals and the number of Q1 individuals collected from cotton plants (*P* < 0.05 in a cumulative hypergeometric distribution test; Table 1); Q1_HW_ were most often found on cucumber, melon and zucchini (all belonging to the family Cucurbitaceae), and Q1_HC_ individuals were significantly over-represented in tomato samplings. Similarly, B_H_ individuals were significantly associated with cotton whereas B_HR_ individuals were associated with eggplant and tomato (both belonging to the family Solanaceae). As in similar surveys (Tsuchida et al., 2002; Brady et al., 2014), the association patterns of biotype-FEC geographical categories overlapped with the crops sampled at each location, where the synchronization between the categories (host plant, geographical location) impeded the stratification of the unique effect of each of these factors. The sampling of Q1-associated FECs from tomato was the only case of a multiple-geographical origin for the sampling of a biotype on a crop in the database (Supplementary Materials). Most Q1 individuals sampled at geographical locations 1 and 4 carried communities HW and H, respectively, in accordance with the general FEC preference at these sites (Table 1). This FEC preference of Q1 individuals was not plant-specific: a majority of HW combinations was observed for eight out of nine sampled crops in region 1; a majority of H combinations was observed for all five crops sampled in region 4. Hence, the symbionts found in Q1 *B. tabaci* collected from tomato point to geographical origin over crop as the key factor associated with specific FECs.

**Table 1:**
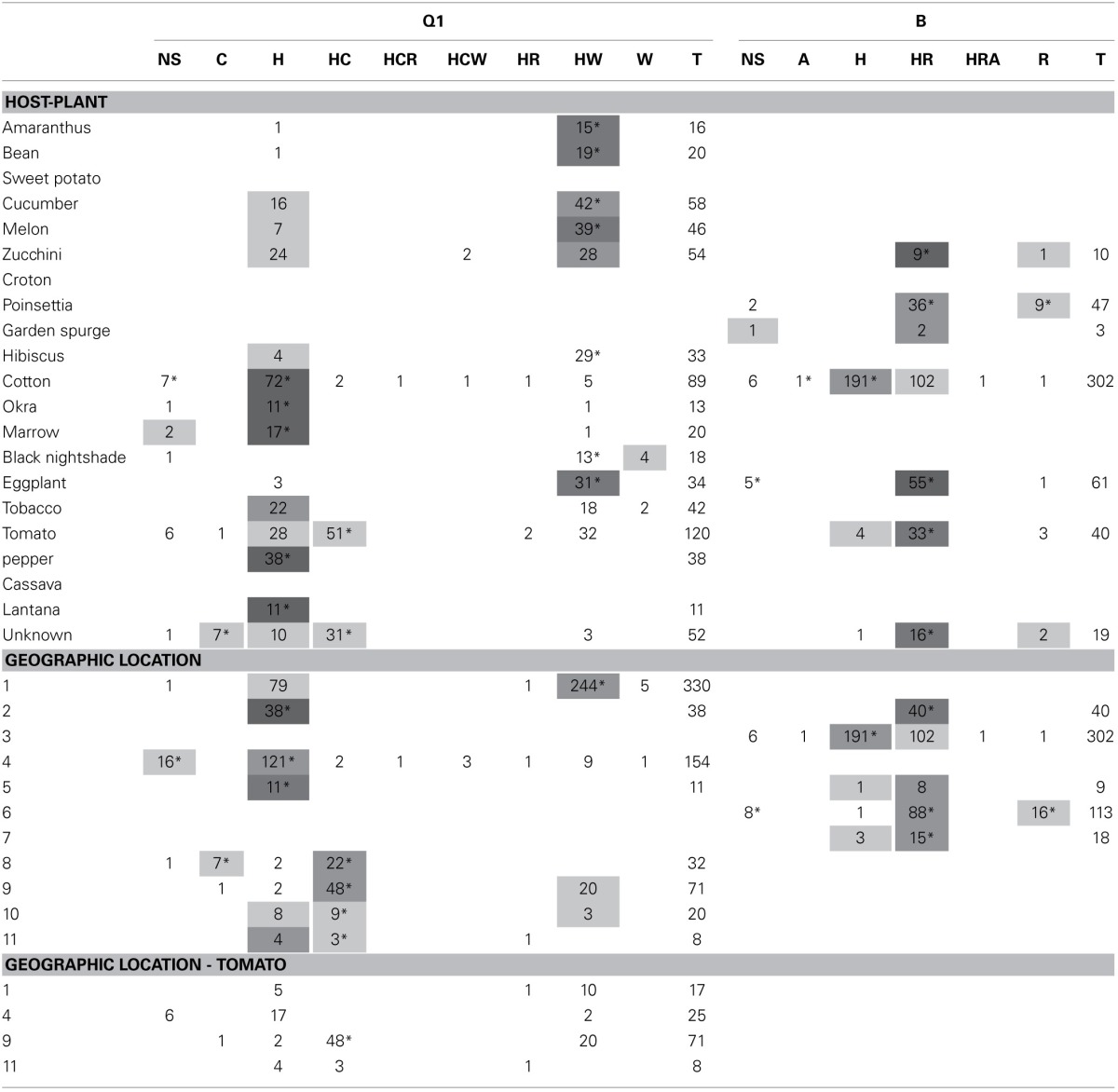
**Distribution pattern of biotype-specific facultative endosymbiont combinations (FECs) across collection sites and crops**.

*Within each cell, numbers indicate the count of individual whiteflies infected with a FEC. Shades of gray indicate the relative abundance of each bacteria within a given sampling category (row) ranging from white (0–10%) to dark gray (90–100%). A, Arsenophonus; C, Cardinium; H, Hamiltonella; R, Rickettsia; W, Wolbachia; NS, no secondary symbionts; T, total*.

^*^Number of individuals from a given biotype-FEC (column) in a category (host plant/collection site; row) is higher than random. Significant enrichment in an ecological category was determined by calculating a cumulative hypergeometric P-value (Methods). Significant cut-offs were determined by setting a False Discovery Rate (FDR) threshold of 10%.

## Discussion

The abundance of facultative endosymbionts has been extensively studied in many arthropods. To the best of our knowledge, the dataset used in this study represents both the largest collection of insects for which communities of facultative endosymbionts have been described and the most comprehensive collection in terms of range of environmental conditions sampled. For example, recent surveys of facultative endosymbionts in whiteflies and aphids are based on approximately 350 and 1100 individuals, respectively (Gueguen et al., 2010; Henry et al., 2013). When compiling such data it is important to bear in mind that biases might be introduced by technical inconsistencies among the independent screening laboratories. In support of the biological relevance of this collection, we verified that all screens were conducted using highly similar protocols and their ability to detect the various endosymbionts is comparable both qualitatively and quantitatively (sensitivity) (Supplementary Material). Here, we used this data to point at environmental and biological factors associated with variations in the diversity and identity of endosymbiont bacteria harbored by whiteflies individuals. Though most insects harbor two or more facultative endosymbionts, the majority of studies focus on the phenotypical significance of an insect–single bacterium association (Zchori-Fein and Bourtzis, 2011). Unlike complex ecosystems such as gut microbiomes, the bacteriome of *B. tabaci* contains a limited number of bacterial members. These include the obligatory endosymbiont as well as an extensively characterized collection of facultative endosymbionts that are repeatedly reported as the key species in this well-defined ecosystem. Hence, basic fingerprinting techniques combined with species-specific sequencing approaches provide a close to full description of the microbial diversity within that niche. This high-coverage description of bacterial genera, allows taking a community view to explore the role of endosymbiont combinations in shaping the distribution patterns of *B. tabaci* individuals. Yet, the presence of screened endosymbionts in low titters (below PCR detectable level) or the occurrence of other bacterial genera cannot be entirely ruled out and might influence the outcome of analysis. First, we observed that the associations are more biotype-specific for multi-member vs. single-member communities. Second, within a biotype, variations in the composition of the symbiotic communities are typically associated with ecological patterns. This is consistent with observations from a smaller-scale global survey of facultative endosymbionts in the cowpea aphid *Aphis craccivora* (Brady et al., 2014) and surveys of facultative endosymbiont populations of the pea aphid *Acyrthosiphon pisum* (Tsuchida et al., 2002; Henry et al., 2013). Third, the analysis revealed a geographical gradient of increasing facultative endosymbiont richness at extreme latitudes. This observation, consistent among highly remote sampling areas, points at the possibility that an evolutionary pressure might be associated with community diversity. A similar increase in complexity correlated with decreasing mean annual temperature and precipitation was previously reported (Tsuchida et al., 2002). Since even two-member multiple infections are suggested to have deleterious effects on fecundity (Oliver et al., 2006), the trade-off between contribution and cost to fitness seems to vary with dependence on climatic factors. Moreover, whereas the geographical distribution preferences of FECs might reflect the historical invasion and spread of the host, the geographical gradient is not composition-specific and is observed independently at a few locations, supporting the role of FEC–insect interactions in shaping the functional repertoire of individuals and hence affecting their pattern of distribution. Finally, the biotype specificity of multiple-member FECs further suggests that the cost of such communities involves highly specific adaptation between host and symbiotic community. These complementary interactions might occur at two different levels: multiple independent host–bacteria interactions and/or interactions between the microorganisms themselves. For example, the complementation of the H combinations with R (HR combinations) is unique to B biotype individuals, whereas the complementation with C or W (HC/HW combinations) is unique to individuals of the Q1 biotype, suggesting a biotype-specific functional adaptation. The scarcity of Q1 individuals carrying both W and C suggests that these genera might have overlapping roles, reducing the likelihood of their co-occurrence.

Complementation of the functional capabilities by insect–symbiont associations is gaining more and more documentation due to the emergence of new genomic sequencing techniques and analytical approaches. In particular, symbiotic microorganisms have been shown to complete partial metabolic pathways in the host, enabling the conductance of otherwise lacking functions (e.g., McCutcheon and Moran, 2010; Sloan and Moran, 2012; Russell et al., 2013). Future exploration of the functional repertoire of different communities, together with the parallel exploration of the functional repertoire in the host insect and host plant, will shed light on the role of mutual complementation in shaping insect ecology. Considering the growing interest in the role of symbiotic interactions in shaping the fitness of plants and animals, the limited size of the communities in this study and the co-localization of their members in a designated organ mark them as a useful model system for such explorations.

### Conflict of interest statement

The authors declare that the research was conducted in the absence of any commercial or financial relationships that could be construed as a potential conflict of interest.
