# Supplementary material for: Variations in the identity and complexity of endosymbiont combinations in whitefly hosts
Source: Front Microbiol. 2014 Jul 4;5:310. doi: 10.3389/fmicb.2014.00310 (PMC4092360; doi:10.3389/fmicb.2014.00310)
Supplement: Supplementary file 1 [file DataSheet1.PDF]

# *Supplementary Material*

# Variations in the identity and complexity of endosymbiont combinations in whitefly hosts

Einat Zchori-Fein[1], Tamar Lahav[2], and Shiri Freilich[2*]

[1]Institute of Plant Protection and [2]Institute of Plant Sciences, The Agricultural Research Organization (ARO), Newe Ya'ar Research Center, Israel.

*** Correspondence:** Shiri Freilich, Institute of Plant Sciences, The Agricultural Research Organization (ARO), Newe Ya'ar Research Center, Ramat Yishay 30095, P.O.B. 1021, Israel. shiri.freilich@gmail.com.

## Supplementary Figures

**Supplementary Figure 1. The biotype distribution of facultative endosymbionts across the six screenings.** Rows represent the six screenings projects reported at the main text. A - Tsagkarakou et al., 2012; B - Bing et al., 2013; C - Zchori-Fein's lab, unpublished. D - Gueguen et al., 2010; E - Thierry et al., 2011; F -Gnankine et al., 2012. Columns represent FSs. A – *Arsenophonus*, C – *Cardinium*, H – *Hamiltonella*, R – *Rickettsia*, W – *Wolbachia*. All facultative symbionts were detected in three of the screenings (B, D, F). The miss identifications of *Arsenophonus* (A) in screening A, *Cardinium* (C) in screening C, and *Wolbachia* (W) in screening E can be related to the absence of the relevant biotypes in the relevant screenings, as can be indicated from the biotype distribution in the columns, pointing at the biotype-endosymbiont associations.

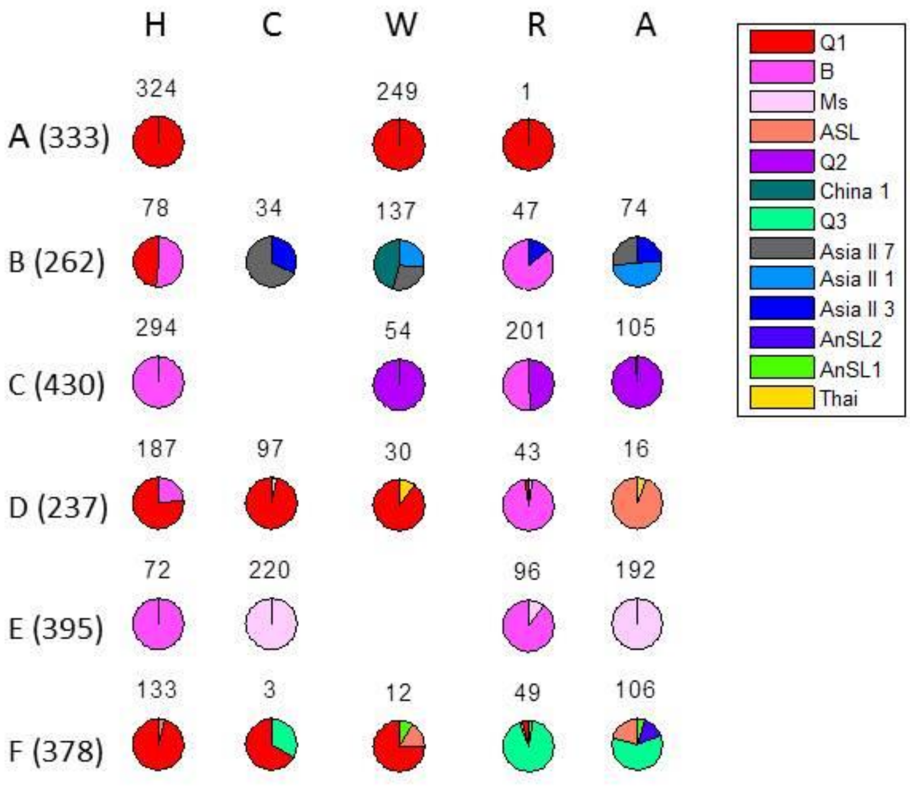

**Supplementary Figure 2. The biotype distribution of facultative endosymbionts.** Numbers in bracts are the numbers of whitefly individuals sampled, carrying a specific facultative endosymbiont. A – *Arsenophonus*, C – *Cardinium*, H – *Hamiltonella*, R – *Rickettsia*, W – *Wolbachia*. Legneds are as in Supplementary Figure 1.

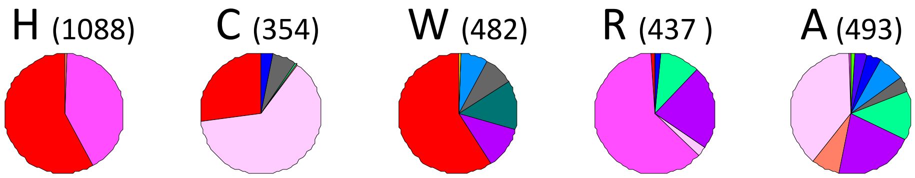


**Supplementary Figure 3.** Mean genera richness of facultative endosymbiont species co-infecting whitefly individuals in each geographic location (Pearson correlation: 0.73, *P*-value 0.05). Circle sizes indicate the number of sampled individuals. The figure repeats the analysis reported at figure 1 in the main text (left), while further dividing the groups within each location according to its contributing project (Project A: green; project B: red; project C: blue; project D: black; project E: dark green; project F: pink). Groups from similar locations that were analyzed by different laboratories show high similarity in their typical genera richness (indicated by gray circles). A gradient of increasing genera richness with the distance from the equator exists between locations that were analyzed by a single group (black circles).

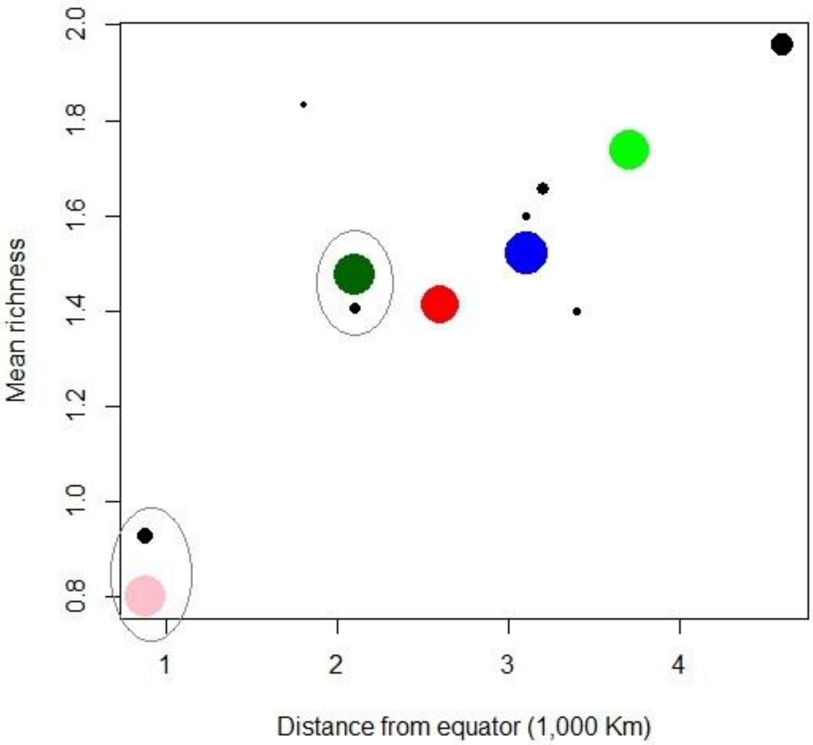

**Supplementary Figure 4. The geographical location distribution of whitefly individuals.** Geographic locations are outlined at Figure 1 in the main text.

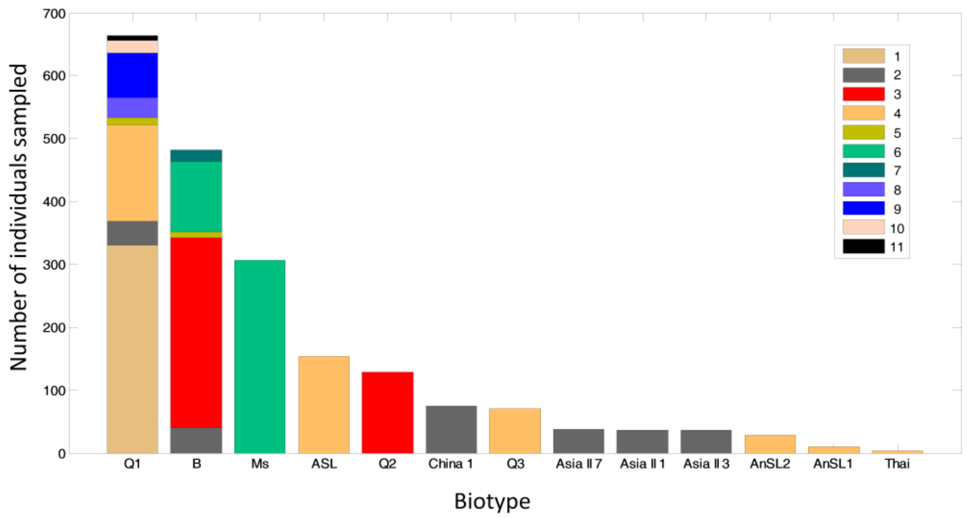


**Supplementary Figure 5. The host-plant distribution of whitefly individuals.**

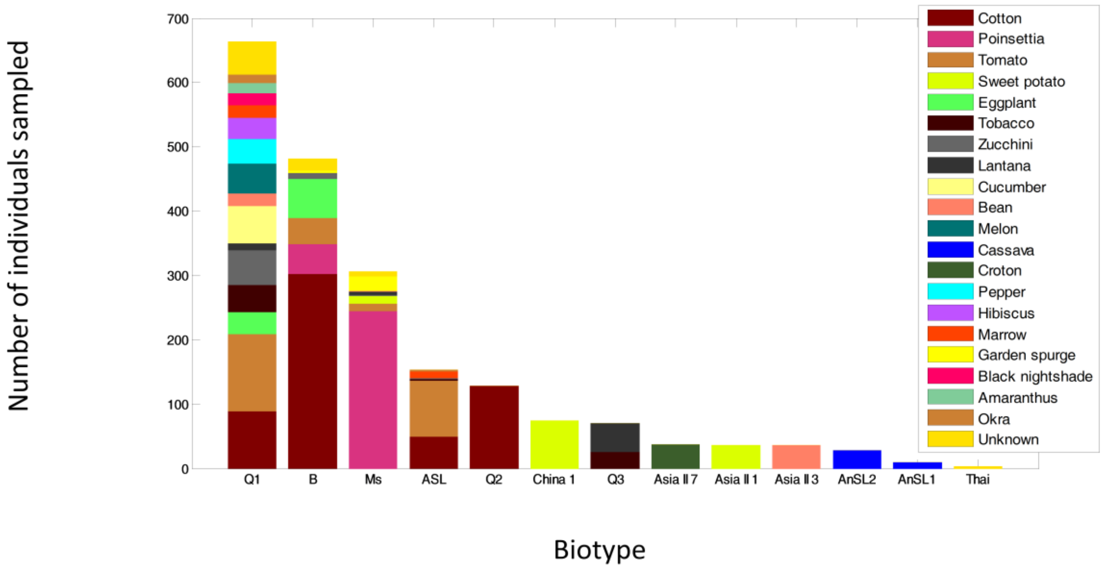

# 1. Supplementary Tables

**Supplementary Table 1.** The PCR protocols used for species detection by the different laboratories included in the screening.

| Contributing labratory | A (Tsagkarakou et al., 20 | B (Bing et al., 2013) | C (Zchori-Fein's lab, unpu | D (Gueguen et al., 2010) | E (Thierry et al., 2011) | F (Gnankine et al., 2012) |
|---|---|---|---|---|---|---|
| DNA extraction | Lysis buffer | Lysis buffer, following | Lysis buffer, following | Extraction with chelex, fo | Lysis buffer (no ref.) | Lysis buffer (no ref.) |
| | similar to Bing but not | De Barro and Driver (199 | Frohlich et al. (1999) | Walsh et al. 1991 | similar to Bing but not | similar to Bing et al. but not |
| | identical | and Frohlich et al. (1999) | | and lysis buffer following | identical | identical |
| | | | | Delatte et al. 2005 | | |
| Primers used to detect | | | | | | |
| symbionts: | | | | | | |
| *Portiera* | | Por-F, Por-R | | Por-F, Por-R | Port-F,Port-R | Por-F, Por-R |
| | | (Zchori-Fein&Brown, 2002) | | (Zchori-Fein&Brown, 200 | (Thierry et al. 2011) | (Zchori-Fein &Brown, 2002) |
| Primers Tm | | 60,58 | | 60,57 | 52 | 58 |
| *Hamiltonella* | Ham-F, Ham-R | Ham-F, Ham-R | Ham-F, Ham-R | Ham-F, Ham-R | Ham-F, Ham-R | Ham-F, Ham-R |
| | (Zchori-Fein&Brown, 200 | (Zchori-Fein&Brown, 200 | (Zchori-Fein&Brown, 200 | (Zchori-Fein&Brown, 200 | (Zchori-Fein&Brown, 200 | (Zchori-Fein&Brown, 2002) |
| Primers Tm | 58 | 60,58 | 58 | 58 | 58 | 58 |
| *Rickettsia* | Rb-F, Rb-R | Rb-F, Rb-R | Rb-F, Rb-R | Rb-F, Rb-R | Rb-F, Rb-R | Rb-F, Rb-R |
| | (Gottlieb et al., 2006) | (Gottlieb et al., 2006) | (Gottlieb et al., 2006) | (Gottlieb et al., 2006) | (Gottlieb et al., 2006) | (Gottlieb et al., 2006) |
| Primers Tm | 58 | 59 | 58 | 58 | 58 | 58 |
| *Wolbachia* | WSP-F,WSP-R | Wol-16S-F, Wol-16S-R | Wol16SF,Wol16SR | 81F, 415R | 81-F,598-R | 81F, 415R |
| | (Zhou et al.,1998) | (Chiel et al., 2007) | (Heddi et al., 1999) | (Braig et al., 1998), (Vaut | (Braig et al. 1998) | (Braig et al. 1998), (Vautrin 20 |
| Primers Tm | 55 | 55 | 55 | 56 | 45 | 56 |
| *Cardinium* | CFB-F,CFB-R | Ch-F, Ch-R | CFB-F,CFB-R | CFB-F,CFB-R | CFB-F,CFB-R | CFB-F,CFB-R |
| | (Weeks et al. 2003) | (Zchori-Fein et al., 2004) | (Weeks et al. 2003) | (Weeks et al., 2003) | (Weeks et al. 2003) | (Weeks et al. 2003) |
| Primers Tm | 56 | 57 | 56 | 56 | 56 | 56 |
| *Arsenophonus* | Ars23S-1,Ars23S-2 | Ars23S-1,Ars23S-2 | Ars23S-1,Ars23S-2 | Ars23S-1,Ars23S-2 | Ars23S-1,Ars23S-2 | Ars23S-1,Ars23S-2 |
| | (Thao & Baumann, 2004 | (Thao&Baumann, 2004) | (Thao & Baumann, 2004) | (Thao&Baumann, 2004) | (Thao & Baumann, 2004) | (Thao & Baumann, 2004) |
| Primers Tm | 60 | 60.5 | 60 | 60 | 60 | 60 |
| *Fritschea bemisiae* | | | U23F, 23SIGR | Frit-F,Frit-R | | Frit-F,Frit-R |
| | | | (Everett et al., 2005) | (Thao et al. 2003) | | (Thao et al. 2003) |
| Primers Tm | | | 60 | 62 | | 60 |

References:


Braig, H.R., Zhou, W., Dobson, S.L. & O'Neill, S.L. (1998) Cloning and characterization of a gene encoding the major surface protein of the bacterial endosymbiont Wolbachia pipientis. *Journal of Bacteriology*, 180, 2373–2378

Chiel, E.,Gottlieb,Y., Zchori-Fein, E., Mozes-Daube,N., Katzir, N., Inbar, M. and Ghanim, M. (2007) Biotype-dependent secondary symbiont communities in sympatric populations of *Bemisia tabaci*. *Bulletin of Entomological Research*, 97, 407– 413.

De Barro, P.J. and Driver, F. (1997) Use of RAPD PCR to distinguish the B biotype from other biotypes of *Bemisia tabaci* (Gennadius) (Hemiptera: Aleyrodidae). *Australian Journal of Entomology*, 36, 149–152.

Delatte H, Reynaud B, Granier M et al. (2005) A new silverleaf-inducing biotype Ms of Bemisia tabaci (Hemiptera: Aleyrodidarum) indigenous to the islands of the south-west Indian ocean. *Bulletin of Entomological Research*, 95, 29–35.

Everett, K.D.E., Thao, M.L., Horn, M., Dyszynski, G.E. & Baumann, P. (2005) Novel chlamydiae in whiteflies and scale insects: endosymbionts ‘Candidatus Fritschea bemisiae’ strain Falk and ‘Candidatus Fritschea eriococci’ strain Elm. *International Journal of Systematic and Evolutionary Microbiology* 55, 1581–1587.

Frohlich, D.R., Torres-Jerez, I., Bedford, I.D., Markham, P.G. and Brown, J.K. (1999) A phylogeographical analysis of the *Bemisia tabaci* species complex based on mitochondrial DNA markers. *Molecular Ecology*, 8, 1683–1691.

Gottlieb, Y., Ghanim, M., Chiel, E., Gerling, D., Portnoy, V., Steinberg, S., Tzuri, G.,Horowitz,A.R., Belausov, E., Mozes-Daube, N., Kontsedalov, S., Gershon, M., Gal, S., Katzir, N. and Zchori-Fein, E. (2006). Identification and localization of a *Rickettsia sp.* in *Bemisia tabaci* (Homoptera: Aleyrodidae). *Appl Environ Microbiol*, 72(5), 3646-52.

Heddi, A., Grenier, A.M., Khatchadourian, C., Charles, H. & Nardon, P. (1999) Four intracellular genomes direct weevil biology: Nuclear, mitochondrial, principal endosymbiont, and Wolbachia. *Proceedings of the National Academy of Sciences USA* 96, 6814–6819.

Thao ML, Baumann L, Hess JM et al. (2003) Phylogenetic evidence for two new insect associated Chlamydia of the family Simkaniaceae. *Current Microbiology*, 47, 46–50.

Thao, M.L. & Baumann, P. (2004) Evidence for multiple acquisition of Arsenophonus by whitefly species (Sternorrhyncha: Aleyrodidae). *Current Microbiology*, 48, 140–144

Vautrin E, Genieys S, Charles S, Vavre F (2008) Do vertically transmitted symbionts co-existing in a single host compete or cooperate? A modelling approach. *Journal of Evolutionary Biology*, 21, 145–161.

Walsh PS, Metzger DA, Higuchi R (1991) Chelex-100 as a medium for simple extraction of DNA for PCR based typing from forensic material. *BioTechniques*, 10, 506–513.

Weeks, A.R., Velten, R. & Stouthamer, R. (2003) Incidence of a new sex-ratio-distorting endosymbiotic bacterium among arthropods. *Proceedings of the Royal Society Series B*, 270, 1857–1865.

Zchori-Fein, E. and Brown, J.K. (2002) Diversity of prokaryotes associated with *Bemisia tabaci* (Gennadius) (Hemiptera: Aleyrodidae). *Annals of the Entomological Society of America*, 95, 711–718.

Zchori-Fein, E., Perlman, S.J.,Kelly, S.E., Katzir,N. and Hunter, M.S. (2004) Characterization of a ‘Bacteroidetes’ symbiont in *Encarsia* wasps (Hymenoptera: Aphelinidae): proposal of ‘*Candidatus* Cardinium hertigii’. *International Journal of Systematic and Evolutionary Microbiology*, 54, 961–968.

Zhou, W., Rousset, F. & O’Neil, S. (1998) Phylogeny and PCRbased classification of Wolbachia strains using wsp gene sequences. *Proceedings of the Royal Society of London, Series B* 265, 509–515.
